# Irish general practitioners' view of perinatal mental health in general practice: a qualitative study

**DOI:** 10.1186/s12875-018-0884-5

**Published:** 2018-12-13

**Authors:** Maria Noonan, Owen Doody, Andrew O’Regan, Julie Jomeen, Rose Galvin

**Affiliations:** 1 Department of Nursing and Midwifery, Faculty of Education & Health Sciences, Health Sciences Building, University of Limerick, Limerick, Ireland; 2 Department of Nursing and Midwifery, Faculty of Education & Health Sciences, Health Sciences Building, University of Limerick, Limerick, Ireland; 3 Graduate Entry Medical School, Faculty of Education & Health Sciences, University of Limerick, Limerick, Ireland; 4 Faculty of Health and Social Care, University of Hull, Hull, UK; 5 School of Allied Health, Faculty of Education & Health Sciences, Health Sciences Building, University of Limerick, Limerick, Ireland

**Keywords:** General practice, General practitioners, Qualitative research, Primary healthcare, Training, Perinatal mental health

## Abstract

**Background:**

Identification of perinatal mental health problems and effective care for women who experience them are important considering the potentially serious impact that they may have on the wellbeing of the woman, her baby, family and wider society. General practitioners (GPs) play a central role in identifying and supporting women and this study aimed to explore GPs' experiences of caring for women with perinatal mental health problems in primary care. The results of this study may provide guidance to inform policy, practice, research and development of curriculum and continuous professional development resources.

**Method:**

In-depth semi-structured interviews were undertaken between March and June 2017 with GPs (*n* = 10) affiliated with a University training programme for general practice in Ireland. Thematic data analysis was guided by Braun and Clarkes (2013) framework.

**Results:**

Data were categorised into three themes with related subthemes: identification of perinatal mental health problems, decision making around perinatal mental health and preparation for a role in perinatal mental health. GPs described the multifaceted nature of their role in supporting women experiencing perinatal mental health issues and responding to complex psychological needs. Inbuilt tools on existing software programmes prompted GPs to ask questions relating to perinatal mental health. Limited access to referral options impacts on assessment and care of women. GPs desire further continuous professional development opportunities delivered in an online format and through monthly meetings and conference sessions.

**Conclusions:**

GPs require access to culturally sensitive; community based perinatal mental health services, translation services and evidence based perinatal psychological interventions. A standardised curriculum on perinatal mental health for trainee GPs needs to be established to ensure consistency across primary care and GP education should incorporate rotations in community and psychiatry placements.

## Background

The perinatal phase, encompassing pregnancy through to the first year after birth, is recognised as a risk period for the development, relapse or recurrence of mental health problems [1, 2]. Women can experience an array of mental health difficulties in this period ranging from depression and anxiety to more serious disorders such as severe depression and psychosis, which are encompassed under the term perinatal mental health problems (PMHPs) [2]. In the UK, population prevalence rates of mental health disorders in early pregnancy has been reported as 27% (95% CI 22–32%), equating to one in four women [3]. Depression and anxiety are the two most common PMHPs that women will experience with prevalence rates in Ireland for antenatal depression at 15.8% [4] and rates for postnatal depression (PND) are reported as 13.2% at six weeks and 9.8% at twelve weeks [5]. Furthermore, perinatal depression and anxiety are frequent co-morbidities [6]. Identification of PMHPs and effective care for women who experience them is important considering the potentially serious impact that they may have on the wellbeing of the woman, her family and wider society [7, 8].

For the majority of women in Ireland, GPs are the first point of contact in the perinatal period and women attend their GP for an initial examination before 12 weeks and thereafter for a set number of antenatal visits [9]. Women will be in contact with a midwife for the booking visit where midwives enquire about emotional issues [9] however, screening for current symptoms of PMHPs using assessment tools is not routinely completed [10]. Postnatal care is primarily provided by Public Health Nurses who screen for PND and anxiety using the Whooley questions and Edinburgh Postnatal Depression Scale and refer to the GP for diagnosis and treatment interventions [9]. Women are entitled to attend their GP for a two week and six-week postnatal check [9] which may be the last routine appointment opportunity for GPs to focus on the mothers’ perinatal wellbeing [11] and offers a safety net to identify poor PMH [12]. Once a PMHP is identified, GPs are usually the first point of contact for women and healthcare professionals (HCPs) and can play a central role in the woman’s recovery through referral to other specialist services, management by monitoring of the woman’s condition or prescribing medication [11].

Current policy and guidance largely overlook the role of GPs in PMH [13]. Few researchers have explored GPs' views and experiences of recognising and differentiating PMHPs and treatment and caring strategies offered by GPs to women [1, 13] with studies generally employing quantitative methods [1]. Furthermore, calls have been made for further research to explore the management of anxiety disorders and to gain an understanding of barriers to disclosure and recognition of PMHPs in practice [1, 14]. Therefore, this study sets out to explore GPs' experiences of caring for women with PMHPs and their views on how best to prepare future GPs for a role in the provision of effective PMH care. To our knowledge, this is the first study to explore GPs role in PMH in the Irish context and is timely in light of the current review of Ireland’s National Mental Health Policy ‘A Vision for Change’ and the publication of ‘Specialist Perinatal Mental Health Services, Model of Care for Ireland’ [15, 16]. The findings of this study may provide guidance to commissioners of PMH services on the support needs of GPs providing care to women who experience PMHPs and their families. The findings also serve to guide development of education and training resources for GPs. The reporting of this study was informed by COREQ criteria [17].

## Methods

An exploratory qualitative design, was employed as its emphasis on context, meaning and experience was considered appropriate to answer the research questions [18, 19]: What are GPs' views, experiences, educational and training needs in supporting women with PMHPs. The University of Limericks, research ethics committee approved the study. All GP tutors affiliated with the Graduate Entry Medical School of that university were contacted by email, provided with information about the study and invited to participate (*N* = 72). Tutors facilitate an early patient contact programme for students and their practices are broadly representative of the national sample of GP tutors. The inclusion criteria were that participants must be registered with the Medical Council of Ireland and have a caseload that includes pregnant and postnatal women.

GPs were purposefully sampled and fifteen GPs responded to the invitation to participate. Recruitment and data collection were undertaken from March 2017 to June 2017 and ceased after ten interviews were conducted as no new information was emerging and data saturation was reached. Within the sample variants were sought relating to GP experience, gender, urban/rural setting, single/group practice and cultural diversity care provision. Characteristics of GPs interviewed (*n* = 10) are provided in Table 1.

**Table 1:** Participant demographic details

Years of experience	Practice Type	Gender
< 5 years (*n* = 1) 5–10 years (*n* = 5) > 25 years (*n* = 4)	Urban (*n* = 7) Rural (*n* = 3)	Female (*n* = 5) Male (*n* = 5)

Interviews were conducted using a pilot tested interview schedule which consisted of open-ended questions derived from the literature [1, 13, 14] and through discussion among the research team which included a practising GP (Table 2). GPs were asked to recall their experiences of providing care to women with PMHPs to ensure that data was representative of their practice. Interviews took place in the university (*n* = 3), GP practice (n = 3) and by phone (*n* = 4) and at a time convenient to the GP. Before interviews commenced participants had an opportunity to ask questions about the study and reflect on participation prior to giving written (face-to-face) or verbal (phone) consent. All interviews were audio recorded with consent, lasted 30–70 mins and were conducted by MN who was unknown to the participants. In addition, after each interview the researcher recorded field notes, reflected on data collection, summarised findings identified emerging codes as data collection and analysis proceeded simultaneously.

**Table 2:** Interview schedule

1. Tell me about your experience of caring for women who experience PMHPs^a^ in primary care? 2. What facilitates you to identify women who may be experiencing psychological distress? 3. What facilitates you to care for women who experience PMHPs^a^? 4. What referral options are available to you in your practice? 5. Based on your experiences how best can a training programme prepare future GPs^b^ for their role in PMH^c^?	

^a^ *PMHPs* perinatal mental health problems, ^b^ *GP* general practitioner, ^c^ *PMH* perinatal mental health

Interviews were transcribed verbatim, anonymised and verified for accuracy by reading transcriptions and listening to recordings concurrently. Braun and Clarkes [19] framework guided thematic data analysis. Codes and themes were discussed, refined and agreed by authors who met regularly to discuss and compare summaries and where interpretation differed, they returned to original transcripts for clarification to reach a consensus about the meaning. Disconfirming evidence was sought and presented in the final analysis. Processes for ensuring data trustworthiness met criteria outlined by COREQ [17]. Methodological rigour was ensured by involvement of three researchers in coding and confirmation of themes, methodological coherence and sampling sufficiency. Furthermore, reflective sessions amongst the research team were undertaken at all stages of the process.

## Results

The data were categorised into three main themes: Identification of PMHPs, decision making around PMH and preparation for a role in PMH (Table 3).

**Table 3:** Overview of themes and subthemes

Identification of PMHPs^a^	• Encountering emotional complexity in general practice • Preconditions for disclosure • Approaches to screening and assessment
Decision making around PMH^b^	• Contrasting referral options • It’s out of my comfort zone
Preparation for a role in PMH^b^	• Luck of the draw GP^c^ training • Continuous professional development opportunities

^a^ *PMHPs* perinatal mental health problems, ^b^*PMH* Perinatal Mental Health, ^c^ *GP* general practitioner;

### Theme 1: Identification of PMHPs

Within the interviews, there was a clear focus on GPs role and support for women experiencing PMHPs. GPs recalled encounters with women who experience PMHPs, identified factors they considered preconditions for disclosure and described their approaches to screening and assessment.

#### Encountering emotional complexity in general practice

PMH was identified as a core component of antenatal and postnatal care offered by GPs who primarily encountered perinatal depression and anxiety in general practice. PND was conceptualised as different to depression that occurs outside of the perinatal period*.*

‘*In a way it has to be looked at separately, women are more vulnerable, they respond differently to medication, things can fluctuate more rapidly, pregnancy itself can bring on a crisis, the delivery itself can bring on a crisis so there are a number of stages and a number of flash point’s' (P6).*

Risk factors were construed by GPs as adverse or vulnerability factors that put women at increased risk of PMHPs. A psychosocial aetiology appeared to be the dominant framework held by GPs when conceptualising anxiety.

*‘Pressure in society, a lot of women are working full time they might have a few kids at home, they have big mortgages, their husband is working a lot of the time and lot of it is maybe triggered by exhaustion or they are just juggling a lot’ (P4).*

Some participants perceived that postnatal anxiety presentation for some women was associated with higher child consultations.

*‘I feel it’s all about a lot of anxiety but it’s packaged up as the child’s problem and it’s hard to disentangle that’ (P2).*

GPs spoke about the difficulties of separating depression and anxiety and the interlink between them.

*‘Two sides of the one kind so when I talk of PND some of that would be more anxiety weighted than depressive symptoms’* (P1).

Severe PMHPs were considered by participants to be rare in general practice and when encountered, the consultation and circumstances left a long- lasting impression with participants ‘*She will stick in my head forever’ (P6).* Participants had limited encounters with women who were suicidal antenatally and when they did were struck by the fact that pregnancy would not have affected her decision.

***‘*** *The fact that she was pregnant didn’t seem to be anything of a deterrent in relation to possibly killing herself which is surprising, I think it was because she was as depressed as she was the world was a bad place and bringing a baby into a bad place was no achievement’ (P9).*

However, they reported encounters with women postnatally with suicidal ideation.

‘*Certainly, they would be a lot of fleeting suicidal thoughts and even a bit of suicidal ideation but I don’t think I have had anyone that had a formal plan’* (P9).

GPs were conscious of a family approach to care and considered that opportunities for engaging men in conversations about their PMH were desired but limited as men rarely accompanied their partners on GP visits. Personal experiences through family, friends and self, increased GP awareness of PMHPs.

*‘It’s more with time and personal experience of friends and self and what pregnancy means and what having children means I guess that you become more aware of it’* (P2).

#### Preconditions for disclosure

Participants identified a number of preconditions that supported disclosure of psychological distress such as women’s openness in relation to PMH issues and suggested that some women were reluctant to disclose their psychological distress particularly women from different ethnic and cultural backgrounds.

*‘There are certain expectations of them within their ethnic group not to have a psychological issue’ (P7)*.

Participants felt that a lack of insight and persistence of stigma were key influencing factors on a woman’s reluctance to disclose her feelings and what was important was being approachable:

*‘It’s a place where they can discuss these things and without necessarily being labelled or admitted or put on medication’ (P1)*.

Participants identified the importance of making time to listen to the woman however, participants also acknowledged that this is dependent on a number of factors:

‘*The art of practice is having that gap or space or vacuum in the consultation where you leave silence … and if you are in listening mode…however it depends on how you say it…it depends on the day, depends on how you are feeling yourself, depends on how the practice is going’ (P5)*.

Time was referred to by all participants as a barrier to engaging in effective PMH conversations.

*‘There is so much emotion around pregnancy and just having time to explore it can be difficult. Hugely time consuming and you can’t really afford as a working-class GP’ (P5).*

GPs were conscious that responding to women experiencing psychological distress may result in longer consultations and this may impact on the time available for other patients presenting with various complexities waiting to see their GP. However, participants also acknowledged that they would make this time for the woman if required.

Building a personal connection was seen as an essential step in developing mutual trust and facilitating women to open up about their psychological distress as was having a good relationship and rapport with the woman. Participants reported that at times there can be a hesitancy to engage in PMH conversations.

‘*Can I be honest with you sometimes I wonder if you really want to open this can of worms and it’s so much easier just to jolly along and check the BP, check the urine, check this and that and have them out the door and see the next patient’* (P5).

‘*There are sometimes and I wouldn’t lie that I would be absolutely hoping that the answer was going to be no problem’ (P7)*.

These feelings for hoping the answer would be ‘no’ were driven by past experiences of dealing with mental health problems, lack of time, resources and referral options to support women.

#### Approaches to screening and assessment

A variety of approaches to assessment and screening for PMH issues were described by participants. Some participants would ask about the woman’s PMH if they felt it might be an issue.

‘*If it’s obvious or if there is a history you would, if it’s volunteered you would and I will be perfectly honest now would you routinely ask about it, theoretically yes in practice maybe, maybe not’ (P5).*

Participants acknowledged that psychological distress in the antenatal period is poorly recognised:

*‘Antenatal care is very protocol driven therefore…it is very easy to ignore the psychological aspect’ (P5).*

In contrast, participants asked women about their PMH routinely as part of the two and six-week postnatal checks:

*‘We would be quite good in fact in asking and it’s probably because of that little reminder on the screen’ (P4).*

However, GPs also acknowledged that the baby can become the focus of the postnatal visits.

*‘I would be guilty of focusing a lot on the child, that’s the big excitement in the room’ (P2).*

Within the assessment process participants primarily referred to*:*

*‘A broad set of questions, you are not just looking at the person but you are looking at the complexities around their family, their environment, and their supports’ (P4).*

Participants referred to ‘*picking up on non-verbal cues’* (P6) and factors they considered to prompt them to engage and assess more:

‘*Your antenna would be raised by people coming clean with you that there is something going on…a history…the usual kind of joy isn’t there, they’re, quiet’ (P5).*

Participants also recalled encounters where women concealed their psychological distress.

*‘Anytime I met her she was pleasant and cheerful as she always was but obviously she could put it on for five or 10 minutes’ (P9).*

GPs described difficulties trying to understand the experience of psychological distress from the perspective of women from ethnic and culturally diverse backgrounds and expressed concerns that they may not be identifying those women’s needs**.**

‘*Very long consultations and just trying to understand where they are coming from culturally can be difficult’ (P1)*.

This left participant’s questioning whether they were seeing the true extent of PMHPs:

‘Are we *only seeing them when it reached boiling point because we [GPs] couldn’t get on top of the problem before that’ (P6).*

In addition, participants identified personal traits or characteristics such as perfectionism as aspects that create greater concern for the woman. Participants referred to using their clinical judgement to identify women experiencing psychological distress and suicide assessment involved a combination of clinical judgement, specific questions and assessment protocols. Within the assessment process participants were guided by information from a variety of sources particularly partners: ‘*It was he who actually told the story’ (P9)*. However, participants also commented that partners may interfere with the woman being able to discuss openly her mental health issues.

‘*I think they were actually stifled in being able to speak and talk and get it out because their partner was always sitting beside her’ (P6).*

Participants acknowledged that they did not formally screen for PMHPs and when screening tools were used it was primarily to confirm a diagnosis, differentiate between anxiety and depression, the co-existence of both and to aid referral decisions. Participants were aware of barriers to assessing PMH such as time, language barriers, fear of *‘making the patient or yourself feel uncomfortable’ (P3)* which led participants to wonder how often they missed psychological distress in general practice*.*

### Theme 2: Decision making around PMH

This theme encompassed the contrasting referral options available to participants which affected assessment, treatment and referral decisions and GPs comfort with pharmacological decisions.

#### Contrasting referral options

Participants described making treatment decisions based on the individual needs of the woman and recommended lifestyle, social and self-management strategies for some women experiencing psychological distress and offered follow up appointments where appropriate. Participants spoke about the complexity of negotiating referrals and while counselling was identified as the main treatment option available to GPs in primary care, public generic counselling services have long waiting times and were considered difficult to access for both the woman and GP:

‘*We have to send the form; the patient has to ring to say did you get the form and I am now confirming that I am going to go and then they get an appointment, for someone who is very distressed and you are asking them to jump through hoops’ (P9).*

Thereby participants’ considered counselling as ‘hit and miss’ and recommended private counselling to women who had financial capacity to access this option.

Three participants identified Cognitive Behavioural Therapy (CBT) as a treatment option however, mother infant mental health interventions and access to psychologists were not available to the majority of participants.

*‘CBT is often indicated as first line treatment but really it’s not an option for a lot of women. They don’t have the means to access it, they don’t have the motivation to access online CBT so it really rules it out as an option’ (P3).*

Participants reported variable access to the multi-disciplinary team including psychiatrists, psychologists and occupational therapists. When participants had access to specialist PMH teams they felt this supported them in their treatment and referral decisions and to provide effective PMH care:

“*We are lucky to have a good support' (P4)*.

The majority of participants relied on the community mental health services however, they did not see such services as the best option for women:

‘*Someone with PMH issues really does not belong in the general psychiatric outpatient clinic.’ (P9).*

A disjoint existed between services where women with severe PMHPs had received care in maternity services and then discharged to community mental health services where all contact with the maternity services ceased.

‘*There should have been a link across the divide…it’s kind of now you’re in the hospital, now you’re out of hospital, now look after yourself and get back to where you were, it wasn’t as cold as that and it wasn’t intended like that, it’s just the way it happened’ (P9).*

Participants identified supports required to enable them to undertake effective decision making in PMH including having access to referral and advise from specialist PMH teams with hospitalisation in a mother and baby unit reserved for severe cases with easy access to interpreter services, free accessible psychological services and preferably ‘*not a drug driven pathway’ (P5).*

#### It’s out of my comfort zone

Participants identified the antenatal period as a particular time when guidance from the psychiatrist was required particularly when considering pharmacological interventions:

‘*Dealing with psychotropic medications and pregnancy is out of my comfort zone’ (P10)*.

One of the main concerns in relation to antenatal pharmacology was teratogenesis and women’s reluctance to take medications. Medication was reserved for women who were not responding to psychological therapies:

‘*If they are not coming through with time as they might want to and sometimes doing all the right things and still there is this dead feeling’ (P8).*

Treatment for women experiencing anxiety focused on providing reassurance, acknowledging the woman’s feelings and in some cases included pharmacological interventions. Participants were explicitly committed to working consensually with women and identified the importance of including women in treatment decisions:

*‘Any decision that would be made around medicine, any kind of management would be made in conjunction with the patient’ (P10).*

Other factors that impacted on participants decision to prescribe medications included their own personal preferences, lack of referral options and women’s reluctance to take medication particularly when breastfeeding. Participants also described the challenges associated with the decision not to prescribe pharmacological interventions:

*‘A challenge sometimes in consultations of any kind where you are not prescribing and you are trying to explain that you are still giving them a good service’ (P1)*.

### Theme 3: Preparation for a role in PMH

This theme comprised aspects relating to the luck of the draw within participants GP training and their engagement in continuous professional development (CPD) opportunities.

#### Luck of the draw GP training

Participants recalled limited information related to PMH as the focus of their specialist training was on obstetric and medical topics and in terms of practical experience:

‘*It can be luck of the draw what you do get exposed to’ (P1)*.

As a result of limited training on PMH in GP training schemes participants were unprepared for their role:

‘*It’s not something I came into medicine having a formula to talk about*.’(P2).

Across interviews participants identified components of what they perceived would be effective training:

‘*Beyond just ticking boxes and try and provide holistic care for women which would include psychological assessment’ (P5).*

Participants also identified the need to prepare trainee GPs for their role in responding to women and their families experiencing psychological distress across cultures in the community setting. Based on their experiences, participants felt that an obstetrics, gynaecology and psychiatric rotation should be compulsory for all trainee GPs which includes a community mental health placement. In addition to support practice and training, participants highlighted that if PMH guidelines were to be developed then it would be *best to distil them into one or two pages (P5)* with *take home messages that are easily remembered (P2)* because appointments are short and GPs need timely access to information. Strategies for facilitating PMH education included input from service users and specialist PMH psychiatrists in GP training programmes.

#### Continuous professional development opportunities

Participants acknowledged that the difficulty for GPs is that they offer a generalist service making it difficult for them to have expertise in all areas including PMH:

‘*You are kind of jack-of-all-trades, you are master of none’* (P5).

Participants reported having limited access to CPD opportunities specific to PMH with pharmacology being identified as a particular ‘*education niche’ (P10)* other suggestions for CPD opportunities included PMH across cultures and paternal PMH. In addition to CPD related to PMH participants also noted a need for further educational opportunities for trainee GPs on looking after their own mental wellbeing:

‘*Those of us in the medical or allied professions can find it harder to admit that we are vulnerable at times’ (P8).*

Participants suggested that CPD could be delivered in the format of an e-learning module on PMH and sessions on PMH at monthly meetings and in the annual GP conference.

## Discussion

### Summary

GPs described the multifaceted nature of their role in supporting women with PMHPs and responding to complex psychological needs. However, meeting the needs of women was challenged at a personal and service level where women are conscious of the social context and expectation placed on them as mothers, while services and referral options available to GPs were limited, under resourced, fragmented and uncoordinated.

### Strengths and limitations

This study used robust and transparent methods to conduct and report the study findings. Participants were from a range of urban and rural population practices, with experience ranging from four to thirty years leading to a varied sample of GPs. An in-depth description of methods, settings and results facilitates judgement of transferability. However, findings should be interpreted in the context of study limitations where the sample was affiliated to one country and participants were from a highly selective group of GPs who might be expected to have a better than average knowledge, especially as they agreed to take part in the study.

### Comparison with existing literature

Participants primarily encountered depression and anxiety in practice, which consistent with previous work, were conceptualised in psychosocial rather than biomedical terms [13]. Participants' perceived that women with anxiety had higher presentations for child concerns and found it difficult to address this when consultations were infant related. This issue is acknowledged in the literature where women with postpartum anxiety were noted to have greater healthcare usage [20] and further research is required to explore the feasibility and process of enquiring about a woman’s PMH at routine and opportunistic child consultations to improve outcomes for women [20].

GPs identified stigma as a barrier to help seeking which is consistent with the literature where women remain silent rather than confront the potential stigma of admitting psychological distress [21]. The taboo around mental health affects HCPs and women alike and may lead to a reluctance in discussing PMH [22]. Button et al. [21] calls for research to identify methods of assessment that elicit women’s confidence in disclosing their feelings combined with increasing HCPs ability to detect psychological distress even when women are avoiding disclosure. Of note in this study was that the integration of PMH questions within GP computer databases reminded participants to enquire about the woman’s mental health and this may be a strategy for implementing routine PMH assessment.

GPs identified cultural and linguistic barriers in their interpersonal encounters with women seeking PMH support. Women from ethnic minority groups may be less likely to consult GPs with PMH concerns and may be particularly disadvantaged in their access to appropriate PMH services because of lack of understanding of the woman’s cultural differences and nuances around PMH [23, 24]. Therefore, in order to improve PMH outcomes for women from ethnic and culturally diverse backgrounds, GPs require access to; translation, interpreting and advocacy services, culturally specific validated assessment tools and education implemented within a system of culturally sensitive and PMH specific referral and support mechanisms [23, 24].

National and international guidance suggests that women with severe PMHPs need to be cared for by services with specialised knowledge and skills [16, 25] and participants in this study reported varied access to specialist and community PMH services. The analysis found that where GPs had limited access to care pathways and referral options including specialist PMH services that this impacted on assessment and treatment decisions. Access to specialist community PMH services has been identified as a key issue for GPs [13]. However, such services are often reserved for women with severe and complex PMH presentations while women with common PMHPs are primarily treated in primary care [26]. Furthermore, women known to services or those with a severe condition are more likely to receive appropriate treatment in comparison to women presenting for the first time with mild to moderate PMHPs [27].

Currently women who seek treatment for PMHPs have limited choice of care pathways and GPs have limited access to proven psychological therapies such as CBT [1, 25] with counselling identified as the main psychological therapy. This is similar to the international context where GPs consider PMHPs in the context of women’s lives and are frustrated at the lack of psychological therapies [1, 13]. Furthermore, GPs described long waiting times for counselling services and this creates a socio-economic divide where women with resources are referred for private counselling whereas those less off have to wait for generic services with long waiting lists. Delays in accessing psychological therapies has been identified as a barrier to help seeking and support [21] and can exacerbate the woman’s psychological distress and direct the GP to pharmacological treatment.

The findings of this study suggest that generally, GPs used pharmacology in the minority of cases and reported a preference for psychological therapies which is in keeping with women’s preferences [21]. However, participants acknowledged that pharmacological interventions were the most appropriate strategy for some women which is congruent with the view that GPs should be more proactive about initiating treatment during the vulnerable perinatal period where severe PMHPs may impact on the child’s emotional and behavioural development [13]. GPs were cognisant of the importance of actively involving women in decisions concerning medication and this acts to enhance the GP service user relationship [28].

Currently no national PMH guidance is available in Ireland and guidelines can offer a resource to enhance GPs' competence and support a consistent approach to education and PMH care provision including pharmacological interventions [29]. Furthermore, implementation of guidelines in practice can prevent against unconscious biases in consultations where GPs selectively collect and interpret evidence from their interactions with women which may lead to an incorrect diagnosis [13]. Guidance may be particularly important for GPs commencing their career in primary care who may lack the breadth of experience to provide effective PMH care [22].

Consistent with the literature, GPs felt that a greater emphasis on PMH in GP training programmes is required [12] and recommended that it would be compulsory for trainee GPs to undertake a psychiatric rotation. Research has found that GPs who had undertaken a psychiatric rotation were significantly more likely to report a higher confidence in their ability to recognise and manage psychiatric presentations in primary care (4.04 vs. 3.70, F = 1.515, *t* = 2.794, *p* < 0.01) [30]. Furthermore, extending rotation options to alternative community based mental health settings has been recommended [31].

Insights gained through education is identified as a key intervention to avoid distorted decision-making processes [13]. Furthermore, PMH training programmes promote consistency in the implementation of evidence-based practices which may result in greater equity in access to PMH services for women and their families [9, 32]. In line with international recommendations education should incorporate resilience training for GPs [31] with further research required to identify strategies to support the mental wellbeing of GPs [33].

Participants suggested that PMH CPD opportunities could be facilitated through e-learning modalities which can be easily completed in the context of current workloads. However, others have argued for CPD to be provided by local PMH specialist teams which would encourage dialogue across professions and shared decision-making [16]. A variety of PMH education strategies have been found to enhance HCPs confidence, knowledge, and practice regardless of education modality [29].

### Implications for research and/or practice

To provide effective PMH care, GPs require timely access to national integrated guidance, culturally sensitive, community based PMH services, translation services, evidence- based perinatal specific psychological interventions and PMH support groups. The integration of validated PMH screening questions to detect a variety of moods within GPs computer data bases may facilitate PMH conversations and assessment. In addition, the coverage of PMH training in a curriculum for trainee GPs needs to be established to ensure consistency across primary care and incorporate rotations in community and psychiatry placements. Research using validated outcome assessment measures is required to examine the effects of such training on GPs practice.

## Conclusion

GPs have an important role to play in supporting women with PMHPs however, they require further support to provide effective PMH care including access to CPD opportunities, specialist PMH and psychological services to meet the needs of increasingly diverse populations. The data and analysis emphasise the context of PMH care provision where GP who have access to specialist PMH services and psychological therapies felt well supported to provide effective care in comparison to GPs who have to negotiate complex referrals with limited access to services.

## Abbreviations

CPD: Continuous Professional Development
GPs: General Practitioners
HCPs: Healthcare Professionals
PMH: Perinatal Mental Health
PMHPs: Perinatal Mental Health Problems
